# Occurrence of Transgenic Feral Alfalfa (*Medicago sativa* subsp. *sativa* L.) in Alfalfa Seed Production Areas in the United States

**DOI:** 10.1371/journal.pone.0143296

**Published:** 2015-12-23

**Authors:** Stephanie L. Greene, Sandya R. Kesoju, Ruth C. Martin, Matthew Kramer

**Affiliations:** 1 Plant and Animal Genetic Resource Preservation Unit, USDA, Agricultural Research Service, Fort Collins, Colorado, United States of America; 2 Irrigated Agriculture Research and Extension Center, Washington State University, Prosser, Washington, United States of America; 3 Forage Seed and Cereal Research Unit, USDA, Agricultural Research Service, Corvallis, Oregon, United States of America; 4 Statistics Group, USDA Agricultural Research Service, Beltsville, Maryland, United States of America; Friedrich-Loeffler-Institute, GERMANY

## Abstract

The potential environmental risks of transgene exposure are not clear for alfalfa (*Medicago sativa* subsp. *sativa*), a perennial crop that is cross-pollinated by insects. We gathered data on feral alfalfa in major alfalfa seed-production areas in the western United States to (1) evaluate evidence that feral transgenic plants spread transgenes and (2) determine environmental and agricultural production factors influencing the location of feral alfalfa, especially transgenic plants. Road verges in Fresno, California; Canyon, Idaho; and Walla Walla, Washington were surveyed in 2011 and 2012 for feral plants, and samples were tested for the CP4 EPSPS protein that conveys resistance to glyphosate. Of 4580 sites surveyed, feral plants were observed at 404 sites. Twenty-seven percent of these sites had transgenic plants. The frequency of sites having transgenic feral plants varied among our study areas. Transgenic plants were found in 32.7%, 21.4.7% and 8.3% of feral plant sites in Fresno, Canyon and Walla Walla, respectively. Spatial analysis suggested that feral populations started independently and tended to cluster in seed and hay production areas, places where seed tended to drop. Significant but low spatial auto correlation suggested that in some instances, plants colonized nearby locations. Neighboring feral plants were frequently within pollinator foraging range; however, further research is needed to confirm transgene flow. Locations of feral plant clusters were not well predicted by environmental and production variables. However, the likelihood of seed spillage during production and transport had predictive value in explaining the occurrence of transgenic feral populations. Our study confirms that genetically engineered alfalfa has dispersed into the environment, and suggests that minimizing seed spillage and eradicating feral alfalfa along road sides would be effective strategies to minimize transgene dispersal.

## Introduction

Two decades have passed since the commercialization of genetically engineered (GE) crops and today over 80% of corn, soybean, canola and cotton grown in the United States are GE varieties [1]. The potential hazards of transgene dispersal have been clearly articulated over the years and include increased invasiveness [2, 3, 4], contaminated genetic resources in centers of crop genetic diversity [5, 6], and adventitious presence (AP) in breeding programs and *ex situ* germplasm collections. Transgene dispersal into feral or volunteer populations may contribute to AP in conventional seed lots [7, 8] or negatively impact weed management practices [9, 10]. Avenues of dispersal can be through seed spillage resulting in admixed seed stocks or transgene flow, defined as the successful movement of GE traits into non GE populations mediated by pollen, seed and clonal propagules [8]. Similar to natural gene flow these avenues vary with species, demographic and environmental attributes [11]. In the last two decades we have seen evidence that transgenes disperse into the environment and that hybridization and introgression occur as well. For example, GE traits have been reported in feral plants of canola and *Brassica rapa—*a closely related weed [12, 13, 14]—refuges of non-Bt cotton [15] and wild *Gossypium hirsutum* in Mexico [16], and feral and wild relatives of creeping bentgrass [17, 18, 19]. Although transgene flow has been confirmed in *Brassica rapa* [13] and *Gossypium hirsutum* [16], introgression has not yet been confirmed in other GE crops [11]. Adventitious presence of GE traits in conventional seed lots has been reported in flax [20, 21], soybean and corn [22], canola [23] and cotton [24, 25]. Although AP can be due to inadvertent commingling of GE and non-GE seed stocks, it can also be a product of hybridization through transgene flow [8].

Alfalfa, the world’s most important forage crop, routinely ranks as a top five crop in terms of economic value and total acreage in the United States [26]. It is the most recent genetically engineered crop to be commercialized. Glyphosate-resistant (GR) alfalfa became available in 2005, and GR alfalfa hay was planted on 80,000 ha, approximately 5% of U.S. seeded acreage, in 2006. In March of 2007, an injunction was passed [27], barring further planting of GR alfalfa. Production was allowed for the life of GR stands; for hay, usually 3–5 years, depending upon location and producer [27, 28], and for seed, two years. With the exception of GE fields planted in 2006 and the fall of 2007, no further source of the transgene was present in the landscape until February of 2011, when GR alfalfa was deregulated a second time.

Because alfalfa is a perennial, insect-pollinated, outcrossing species, the potential for gene flow has been widely recognized [29, 30]. Since 2005, industry (e.g. the National Alfalfa and Forage Alliance (NAFA), and the Association of Seed Certifying Agencies (AOSCA)) has focused on developing and implementing formal strategies to ensure AP-sensitive producers are not adversely impacted by GE trait escape [31, 32, 33]. The intent of these strategies is to support coexistence of GE and non-GE alfalfa producers, not to restrict the distribution of the transgene into the environment. A common feature in all alfalfa coexistence management practices is the control of feral alfalfa around seed production fields.

The occurrence of feral alfalfa in areas that grow alfalfa is widely recognized. In this paper we expand on the definition of ferality proposed by Bagavathiannan and Van Acker [34], namely, “individuals of a cultivated crop that survive, reproduce successfully and establish a self-perpetuating population in either a natural or semi-natural habitat,” to better reflect the industry definition, which includes individual plants and colonies outside of cultivated fields, that may or may not be self-perpetuating. With the exception of introduced naturalized populations of yellow flowered alfalfa (*Medicago sativa* subsp. *falcata* L.), reported in 27 U.S. states and Canada [35], close relatives of alfalfa do not occur in North America [30]. However, feral alfalfa is commonly found along road ways and disturbed habitats [36]. In a survey of 940 roadside sites in 47 counties in California, Idaho, Pennsylvania, South Dakota and Wisconsin, approximately 22% of the sites had feral alfalfa populations within 2000 m of cultivated alfalfa [37]. A survey in southern Manitoba concluded that feral plant occurrence was great enough to warrant management to effectively confine transgene movement [38]. Although alfalfa feral populations contain relatively few plants compared to field stands, their positive contribution to local gene flow has been suggested using simulated individual trap plants [39]. Although both non-GE and GE feral alfalfa plants can potentially compromise varietal purity, transgenic feral plants could contaminate AP-sensitive seed lots (e.g. export or organic seed). Glyphosate resistant GE feral plants may increase through selection if glyphosate-only regimes are used in weed management, and may necessitate the need to change these practices. Since the initial release of GE crops, reports have confirmed that transgene dispersal into the environment can occur in most GE crops, but to varying degrees, due to attributes that influence seed spillage and seed- and pollen-mediated gene flow such as mating system, propagule dispersal dynamics and the occurrence of hybridization partners (i.e. feral, volunteer or wild relative populations). The short duration of the first deregulation period of glyphosate resistant alfalfa provided an opportunity to assess transgene penetration into feral populations, since it provided a limited pulse of the transgene on the landscape. Previous studies have documented the occurrence of feral alfalfa and its potential to contribute to local gene flow. However, since deregulation, no studies have quantified the dispersal of the alfalfa GR transgene outside of cultivated fields. There is limited information regarding the risk that transgenic feral plants serve as reservoirs or conduits that might facilitate the movement of transgenes into conventional alfalfa and there is minimal information on how environment and agricultural production may influence alfalfa feral/transgenic feral plant occurrence. Our objectives were to (1) evaluate evidence that feral transgenic plants could spread transgenes to fields of non-GE plants and (2) determine environmental and agricultural production factors influencing the location of feral alfalfa, especially transgenic plants.

## Materials and Methods

### Study area and survey sampling design

Alfalfa seed is produced mainly in the western United States, with intensive production located in Fresno County, California; Canyon County, Idaho; and Walla Walla County, Washington. Study areas were located within these three counties, and encompassed areas where alfalfa seed is produced (Table 1). Since survey and collecting efforts focused on public rights of way, no specific permission for access was required, nor was a collecting permit needed to sample feral alfalfa plants, since they are not a protected species. We generated a probability-based, spatially balanced random survey design using the Geostatistical Analyst, Spatially Balanced Points tool in Arc GIS 10.0 (Esri Inc., Redlands, CA). Spatially balanced designs compute a set of sample points that result in Voronoi polygons with a similar area, thus maximizing spatial independence among sample locations and providing more statistical efficiency [40, 41]. The Spatially Balanced Points tool also provides a way to vary sampling intensity by using relative inclusion probabilities, which specify the probability that a location will be selected relative to other locations. We used this in Fresno County to focus the survey on areas where alfalfa was grown. Input data included a maximum bounding rectangle for the survey area, the sampling frame, which consisted of a road layer with major highways and roadways within populated places removed, and an inclusion probability layer. The resolution for survey locations was set to 10 m, which corresponds to the general resolution of a car GPS. Depending on the study area, 700–800 random locations were generated.

**Table 1:** Location, size, climate, and crop characteristics of three areas in the western United States surveyed for feral alfalfa.

State	County	Geographic coordinates (center)	Study area (km^2^)	Ann.ave. temp. (°C)	Ann.ave. precip. (mm)	No. of historic GE seed fields	Total area historic GE seed fields (ha)	Commercial alfalfa pollinator	Major crops
CA	Fresno	36.6039°-120.0967°	1571	13	180	1	64	Honey, Leaf cutter	Alfalfa, winter wheat, cotton, almonds, grapes
ID	Canyon	43.6087°-116.7057°	1564	11	210	51	799	Leaf cutter	Grass/pasture, alfalfa, corn, winter wheat, dry beans
WA	Walla Walla	46.1894°-118.5560°	1786	11	330	14	625	Leaf cutter, Alkali	Winter wheat, grass/pasture, alfalfa, spring wheat, potatoes

### Field survey

The surveys were conducted in August and September of 2011, five to six months after GE alfalfa was deregulated a second time. A small area in Fresno County was resampled in May of 2012 to provide the same sampling density as Walla Walla and Canyon counties. Since alfalfa seed is frequently planted in the fall and seedlings less than 12 months old can be identified, we felt confident that almost all roadside plants were established prior to the second deregulation, and that we could identify roadside seedlings originating from newly planted alfalfa fields. Routes were constructed by subdividing 40–60 adjacent locations, and uploading the coordinates as custom POI files into a GPS device (Garmin nuvi 220, Garmin Intl., Olathe, KS). The Garmin route optimization tool was then run and the GPS was used to navigate to each site. It quickly became obvious that roadside feral plants were rare, so we stopped whenever we saw feral populations and included these as found locations. At each location we collected data from both sides of the road and considered each side as a separate data point. The area surveyed consisted of a rectangle with a 30 m length oriented parallel to the road way, and a width reflecting the distance between the county road surface and adjacent private property. GPS coordinates were taken at the center roadside edge of each site. Presence/absence of feral alfalfa plants, population size (counted up to 100, visually estimated after that), age class (young, old, mixed) and environmental characteristics were recorded at each survey site (Table 2). Duplicate samples representing tissue from the same plants were obtained to support transgene testing using two different methods. Four fully expanded green leaflets were sampled for each individual plant and two leaflets per plant were placed in a single envelop (x2), and pooled with leaflets sampled from up to ten individual plants, since we could detect the transgene from a pooled sample containing one positive plant and nine negative plants). One set of samples was air dried; the second set was kept at approximately 4°C until lyophilized in a VirTis Freeze Mobile 24 instrument (Gardiner, NY) for DNA isolation. At sites with a large number of feral plants, the sample area was divided into thirds, and leaf samples from 10 random plants were collected from each subdivision for a total of 30 plants (three pooled leaf samples from 10 plants each). If seed was present, pods from five racemes per plant were collected from up to 10 random plants and pooled. Large populations were subdivided and sampled in the same way as leaf samples. Leaf and seed samples were obtained from the same plants, which allowed us to assess current year gene flow (i.e. current year transgene pollen transfer would be suggested if all plants were negative but seed samples were positive). Note, however, that since a seed sample represents a large population of individual plants, a positive seed sample result is more likely than a positive leaf sample, depending on the number of contributing individuals to each of the pooled samples. In 2013 we conducted demographic surveys on 10 feral populations in Fresno and Canyon counties to better understand population dynamics, including persistence. Individual plants were tagged and leaves sampled and tested for the presence of the transgene. The same data were collected on these populations.

**Table 2:** Variables used in generalized linear model explain the occurrence of feral populations and transgenic feral populations growing along rural road verges.

Variable	Description	Source
Crop Adjacent	1 = Wild/Ruderal, 2 = Orchard, 3 = Forage,4 = Row Crop, 5 = Other	Collected by authors
Crop Ahead	1 = Wild/Ruderal, 2 = Orchard, 3 = Forage, 4 = Row Crop, 5 = Other	Collected by authors
Crop Behind	1 = Wild/Ruderal, 2 = Orchard, 3 = Forage,4 = Row Crop, 5 = Other	Collected by authors
Vegetation Management	1 = Burned/graded/mowed, 2 = Sprayed,3 = Tilled	Collected by authors
Vegetation Cover	1 = Bare, 2 = Continuous, 3 = Patchy	Collected by authors
Vegetation Height	1 = Short, 2 = Medium, 3 = Tall	Collected by authors
Species Diversity	1 = High, 2 = Medium, 3 = Low	Collected by authors
Elevation (m), Slope (deg), Aspect (deg)	30 x 30 m spatial resolution	USGS National Elevation Dataset [57,58]
Precipitation (mm)	Seasonal average from 2005 to 2012 (obtained as 30 arc second spatial resolution; monthly temporal resolution)	PRISM Climate Group, Oregon State University, Available at: http://prism.oregonstate.edu (accessed 2/12/2013; verified 10/20/2013.
Temperature (° C)	Minimum and maximum seasonal average from 2005 to 2012 (obtained as 30 arc second spatial resolution; monthly temporal resolution)	PRISM Climate Group, Oregon State University, Available at: http://prism.oregonstate.edu (accessed 2/12/2013; verified 10/20/2013.
Proximity	To closest alfalfa production area. 1 = < 2000 m field; 2 = >2000 m, but within production area; 3 = bordering production area (5000 m); 4 = outside of production area > 5000 m)	USDA National Agricultural Statistics Service Cropland Data Layer. 2013. Published crop-specific data layer [Online]. Available at http://nassgeodata.gmu.edu/CropScape/ (accessed 2/12/2013; verified 10/20/2013. USDA-NASS, Washington, DC.
Transport Spillage	Potential for spillage during transport. 1 = High likelihood (main road); 2 = Medium (secondary road), 3 = Low (tertiary road, mainly local traffic), 4 = Very Low (gravel road, only local traffic)	Google Earth and ArcGIS 10.2
Spillage	Potential for spillage during production and transport. 1 = Very high (adjacent to historic seed field/ or along main route to seed conditioning plant); 2 = High (close to historic field or along secondary road to plant); 3 = Medium (within seed production area or along road close to transport route); Low (Outside of seed production area or secondary/gravel road isolated from transport route	Google Earth and ArcGIS 10.2
Historic Seed Field Location	Euclidean distance from survey location to closest historic GE seed field	Geographic coordinates of GE seed fields grown during the first deregulated period provided by Forage Genetics International (Nampa Idaho)
Historic Hay Field Location	Distance class to the closest historic GE hay field was provided for 192 survey locations where feral plants were observed. Distance classes: < 1.6 km, 1.6–8 km; 8–16 km, > 16km. Remaining locations were classified based on proximity to buffer zones placed around the 192 locations, starting at <1.6 km and working outward	Monsanto Inc. (St. Louis, MO)

### Testing for the transgene

The gene encoding the herbicide tolerant form of the 5-enolpyruvylshikimate-3-phosphate synthase (EPSPS) from *Agrobacterium tumefaciens* strain CP4 has been engineered into alfalfa to produce the CP4 EPSPS protein that conveys resistance to glyphosate [42]. Air dried leaf samples and seed were tested for the presence of the protein produced by the transgene using lateral flow AgraStrip® RUR Seed and Leaf TraitChek™ test strips. (Romer Labs Inc, St Louis, MO, USA). The method provides a qualitative threshold test based on CP4 EPSPS-specific antibodies coupled to a color reagent. Watrud et al. [43] compared the reliability of immunological lateral flow test strips to PCR and found they were 100% accurate.

Dried leaf tissue was crushed and placed in a 1.5 ml Eppendorf tube with 0.5 ml distilled water. The slurry was stirred using a disposable stirrer, and the TraitChek™ test strip placed in the tube. After 5 min, samples were scored as either positive or negative for the transgene based on the presence or absence of a colored test line. Samples were processed in groups of 18 with a positive and negative control sample included in each group. Sensitivity of AgraStrip® RUR Seed and Leaf TraitChek™ test strips is one seed in 600, so 600 seeds were tested at a time. To facilitate testing, we pooled seed collected from each sample population. Twenty-five seeds were weighed and this weight was used to estimate the weight of 600 seeds. Six hundred seeds were ground for 5 pulses of 10 seconds each, using a spice grinder (Cuisinart, East Windsor, NJ) and a separate bowl was used for each sample to avoid cross contamination. Ground seed was placed in 15 ml centrifuge tubes, and 4 ml distilled water added. The tube was shaken for 15 seconds, allowed to settle for 1 minute, and TraitChek™ test strip placed in the tube. After 15 min, the sample was scored as either positive or negative for the transgene based on the presence of a colored test line. All seed collected from each population was tested.

### Quantitative PCR confirmation of TraitChek™ test strip positive samples

Genomic DNA was extracted from the lyophilized duplicate leaf samples of those that tested positive for the transgene by TraitChek™ test strip. If there were greater than 10 leaflets in an envelope, leaflets were divided into several tubes and labeled Site # A, B, C. When extracting DNA, we started with one tube and if that was positive, we did not isolate DNA from the remaining samples. Genomic DNA was initially purified from pulverized freeze-dried leaf tissue using the Rapid One-Step Extraction (ROSE) method [44]. The lyophilized leaf samples were placed in 2 ml grinding tubes (Daigger BIO4050; CA) with three grinding beads (5/32” Craig Ball Sales, DE) and ground for 1 min at 1100 strokes/min in a genogrinder (SPEX SamplePrep, NJ). Samples were checked for complete grinding, and ground for another min if necessary. Tubes were centrifuged at 10000 x g for 5 min to pellet the lyophilized tissue, prior to adding between 0.8 ml (1–2 leaflets) and 1500 ml (3–5 leaflets) of ROSE buffer (10 mM Tris pH 8.0, 312.2 mM ethylenediaminetetraacetic acid, 1% sodium lauryl sarkosyl, 1% polyvinylpolypyrrolidone, with 1% beta-mercaptoethanol added just prior to use) to each tube. The samples were thoroughly mixed until all plant material was suspended in the buffer, and then incubated at 90°C for 20 min, inverting the samples every 5–10 min. Samples were placed on ice for 10 min and then centrifuged at 12000 x g for 5 min. The supernatant was diluted 150 fold with sterile-double distilled water and 2 μl of this dilution was used in a 20 μl reaction for either real time PCR (qPCR) or PCR. Primer and probe sequences for PCR and qPCR were provided by Monsanto (confidential information). For qPCR, the reaction mix consisted of ~ 1 unit of Biolase DNA Polymerase (Bioline USA; Taunton MA) per 20 μl reaction, 2.0 μl of 10x NH_4_ reaction buffer, 2 μl DNA (or sterile distilled water for negative control), and final concentrations of 0.2 mM dNTPs, 500 nM primers, 250 nM probes, and 2.5 mM MgCl_2_. Samples were run on a BioRad C1000 Touch™ Thermal Cycler (Bio-Rad, Hercules, CA) and analyzed on a CFX96 Touch™ Real-Time PCR Detection System (Biorad, Hercules, CA). The program consisted of one cycle of 95°C for 3 min, followed by 45 cycles of 95°C for 15 sec, 60°C for 1 min (data taken at this step). Results were recorded as positive or negative; no quantification was attempted. For samples analyzed by PCR, the reaction mix was the same, but in this case each event was analyzed in a separate tube and there was no probe added. Products were separated on a 2% TAE (Tris, Acetate, EDTA) gel and examined for the presence of event specific bands. Negative samples were further purified by isopropanol precipitation according to the original protocol [44] and rerun.

### Data analysis

#### Spatial analysis

The occurrence of feral plants and transgenic feral plants was reported as presence/absence data. At locations where multiple samples were collected due to large population sizes, the location was scored as present for the transgene if one or more samples were positive. No effort was made to quantify transgene presence within individual populations. To analyze spatial patterns and explore spatial clustering of feral populations, nearest neighbor and hot spot analysis were performed using spatial statistics tools in ArcGIS 10.2 (ESRI, Redlands, CA). As a first step, we tested the null hypothesis that feral plants were randomly distributed throughout our study areas using nearest neighbor analysis. Nearest neighbor analysis was performed using the average nearest neighbor function in ArcGIS to evaluate the degree of clustering. The analysis calculates the ratio of observed average nearest neighbor distance and expected average distance based on random distribution. If the ratio differs significantly from zero, the null hypothesis is rejected and the spatial pattern is either clustered (<1) or over-dispersed (>1) [45]. We created separate data layers for each of the three study areas, which included feral site locations (latitude and longitude) and performed nearest neighbor analysis on each of the study areas. Pinpointing the location of clusters can help identify what causes clusters. We used Optimized Hot Spot Analysis (HSA), a spatial cluster detection method to see if feral populations tended to cluster in alfalfa seed and hay production areas. Seed production areas were based on buffering historic seed field locations, and hay production areas were based on the USDA National Agricultural Statistics Service Cropland Data Layer. Classifying feral locations as either 1 or 2 was based on their occurrence in a seed production area (1), or non-seed production area (2). HSA uses the Getis-Ord Gi* statistic [46] to identify significant clusters by computing the sum of values for a site and its neighbors, and compares this value to the sum of values for all sites. The Getis-Ord local statistic was used to determine which locations were spatially clustered into seed production and non-seed production areas. The False Discovery Rate Correction was applied to adjust the statistical significance to account for multiple testing and spatial dependence.

#### Statistical analysis

Table 2 provides definitions for the candidate independent variables used in our analysis. A generalized linear model (GLM- binomial family) was used to determine if a relationship existed between these qualitative and quantitative variables and feral plant occurrence from random survey sites using the R software (http://www.r-project.org/). The ‘step’ function, coupled with occasional subjective judgment, was used for variable selection. The assumption underlying this modeling is that the presence of feral alfalfa can be predicted in part by general ecological variables, the kinds of variables that influence the distribution of plant species as well as agricultural variables. GLM was also used to explore the relationship between explanatory variables and the occurrence of transgenic feral plants. Here, we were interested if the independent variables we had could predict where the occurrence of transgenic plants differed from that of non-transgenic plants. An example of such a factor is proximity to a field previously planted in transgenic alfalfa. Tests for partial autocorrelation and clustering were performed using the EVariogram function from the CompRandFld 1.03 R package [47] to fit variograms for binary data that described the degree of spatial dependence of feral populations within each study area. The data were visualized with lerolograms, which plot autocovariance for binomial data as a function of distance. Autocorrelation was modelled using the FitComposite function, with an exponential correlation model (autocorrelaton decays as an exponentially decreasing function with increasing distance). All (both random and found) survey locations within a county were used for this analysis.

## Results

**Fig 1:**
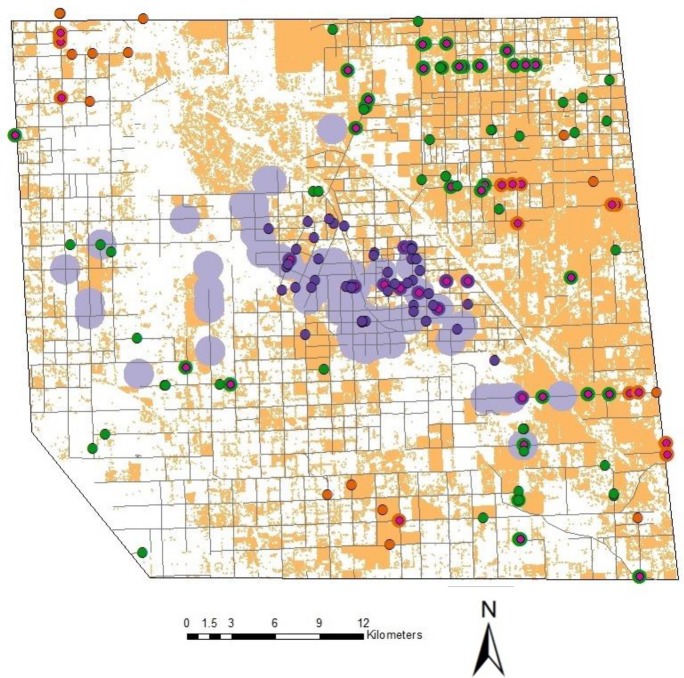
Distribution of roadside feral alfalfa plants in Fresno County, California. Hot spot analysis showed significant clustering of roadside feral populations (dark purple, dark orange) in alfalfa-seed (purple) and hay-production (orange) areas. Non-clustering populations are also evident (green). Transgenic feral populations (pink) occur in seed- and hay-production areas, as well as along major roads used to transport seed.

**Fig 2:**
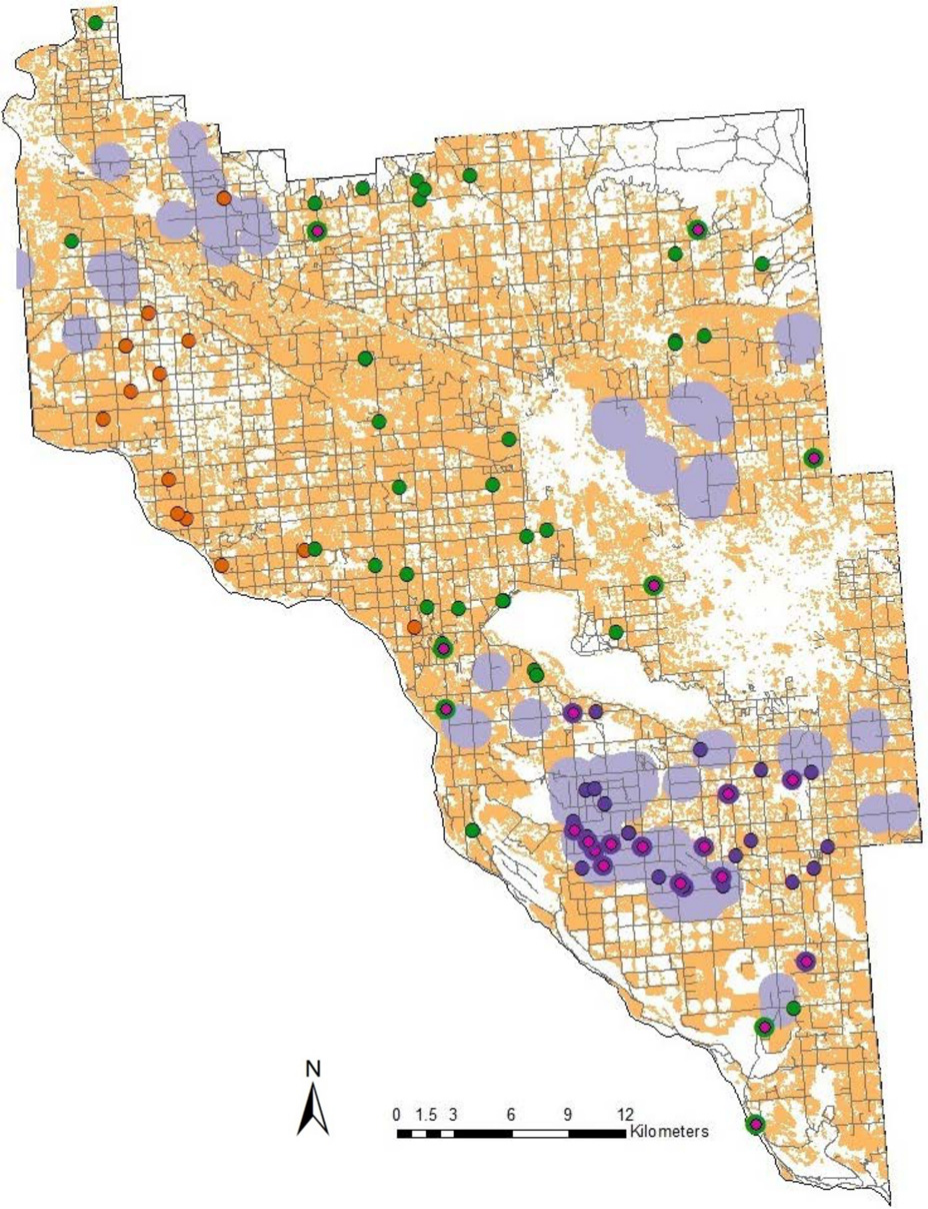
Distribution of roadside feral alfalfa plants in Canyon County, Idaho. Hot spot analysis showed significant clustering of roadside feral populations (dark purple, dark orange) in alfalfa-seed (purple) and hay-production (orange) areas. Non-clustering populations are also evident (green). Transgenic feral populations (pink) occur mainly in seed production areas.

**Fig 3:**
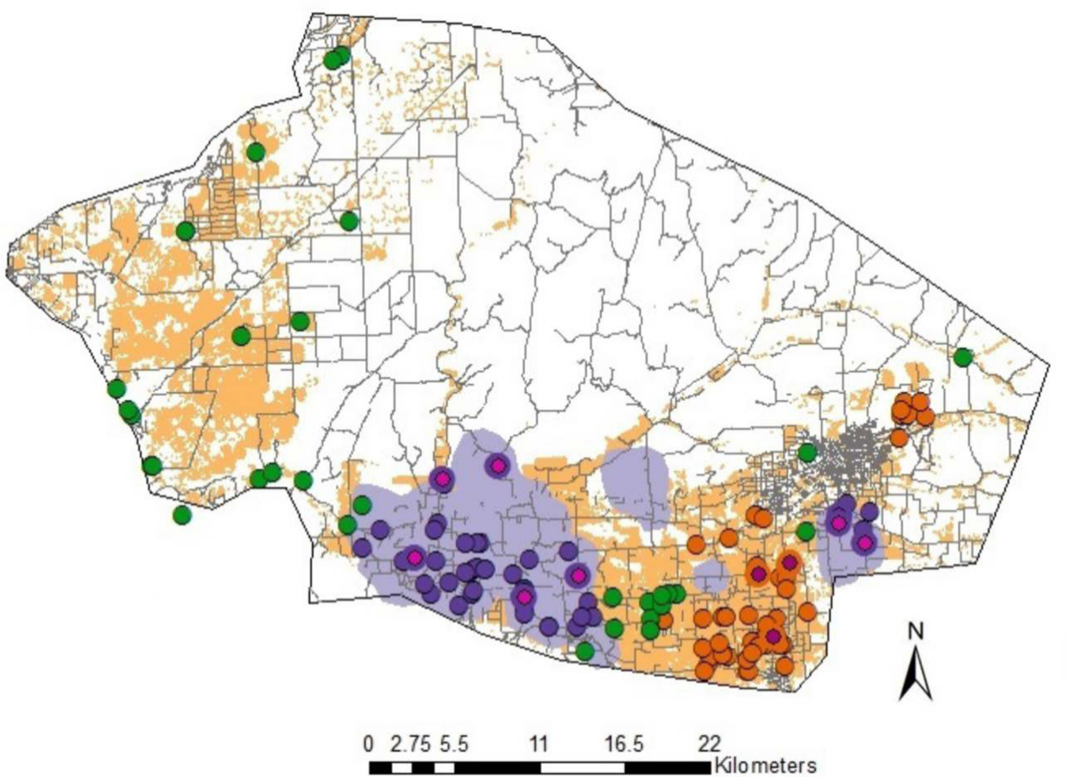
Distribution of roadside feral alfalfa plants in Walla Walla County, Washington. Hot spot analysis showed significant clustering of roadside feral populations (dark purple, dark orange) in alfalfa-seed (purple) and hay-production (orange) areas. Non-clustering populations are also evident (green). Transgenic feral populations (pink) were clustered in seed and hay production areas.

In total we travelled 6000 km of rural roads in three alfalfa-seed-production areas in the western United States, stopping at 4,190 random sites to survey the occurrence of feral alfalfa plants and observed 185 sites with feral plants. We surveyed an additional 390 found sites and observed feral plants at 219 of these sites. Table 3 summarizes the results. Across study areas, 4.4% of our random sites contained feral populations. The greatest number of sites with feral plants was observed in Fresno County, California and the fewest in Canyon County, Idaho. Size of the feral populations varied, with the largest populations in Canyon County and the smallest in Walla Walla County. Forty-eight percent of the feral populations consisted of mixed age classes. In all three counties, average nearest neighbor analysis rejected the hypothesis that feral plants were randomly distributed. The observed mean distance between nearest neighbors was 845 m, 1318 m and 812 m in Fresno, Canyon and Walla Walla counties, respectively. The nearest neighbor ratio was 0.53, 0.57 and 0.49 for Fresno, Canyon and Walla Walla counties; these values suggest that sites are clustered. Significant but low spatial autocorrelation was found in all three study areas and there was little spatial autocorrelation past 200 m. This varied somewhat by study area, with autocorrelation in Fresno County extending to an estimated 190 m and Canyon and Walla Walla counties extending to 70 m and 82 m, respectively. Significant autocorrelation is consistent with the results of the nearest neighbor analysis that suggested observations were clustered. Significant clustering occurred in both seed production and non-seed-production areas. Clusters in non-seed-production areas tended to occur in hay-production areas. Figs 1–3 show the results of the analysis for Fresno, Canyon and Walla Walla, respectively. We interpret these spatial analyses as suggesting that many or most of the sites where feral plants occurred were of independent origin, but tended to cluster in seed and hay production areas because those are places where seeds tend to get dropped. Significant but low spatial autocorrelation suggested that plants on some sites may colonize nearby sites.

**Table 3:** Number of non-GE (-) and GE feral (+) populations observed at random and found sites in three study areas.

Area	Kmsurveyed	Random sites			Found sites			TotalFeral km^-1^
		Total	(-) feral	(+) feral	Total	(-) feral	(+) feral	
Fresno	1596	1416	37	18	200	59	55	0.11
Canyon	2539	1350	55	15	48	18	9	0.04
Walla Walla	1834	1424	55	5	142	71	7	0.08
	5969	4190	147	38	390	148	71	

Results for the stepwise logistic regression model presented in Table 4 support our spatial analysis. In all three counties, the variables, Crops Adjacent and Transport were significant. Road Verge, Vegetation Cover, and Species Diversity were significant for Fresno and Canyon counties, and Proximity was significant in both Fresno and Walla Walla counties. Although we found significant explanatory variables, this needs to be taken in context. A model which assigned the status of all sites the most common category, (i.e. feral plants are absent) would be wrong 4.05% of the time (i.e. the overall error rate is 0.0405), as most random sites did not have alfalfa. The error rates for the regression models (i.e. the probability that the site had plants was greater than 0.5 when the site had no plants, or the probability was less than 0.5 when the site did have plants) were hardly different than 0.0405; 0.038, 0.049 and 0.041, for Fresno, Canyon and Walla Walla counties, respectively. In practical terms, other than spatial clustering of sites near hay and seed production areas, described above, locations of clusters were not well predicted by the variables we had available and fortuitous events probably underlie many of the alfalfa colonization occurrences.

**Table 4:** Stepwise logistic regression model for the influence of alfalfa production area, cropping pattern, roadside verge characteristics, transport spillage and climate on the occurrence of roadside alfalfa populations.

Parameter	df	Deviance Residual	Df Residual	Deviance	Pr(>Chi)
**Fresno**					
NULL			1406	477.16	
Proximity	3	24.301	1403	452.86	0.00002
Aspect	1	6.877	1402	445.98	0.00873
Crops Adjacent	3	17.207	1399	428.78	0.00064
Crops Behind	4	20.069	1395	408.71	0.00048
Veg Cover	3	32.340	1392	376.37	0.0000004
Species Diversity	3	18.608	1389	357.76	0.000329
Transport	3	41.672	1386	316.09	0.000000003
Fall Precip	1	10.511	1385	305.58	0.00118
**Canyon**					
NULL			1330	560.08	
Crops Adjacent	5	19.026	1325	541.06	0.001901
Veg Cover	3	15.610	1322	525.45	0.001363
Species Diversity	3	10.107	1319	515.34	0.017677
Transport	3	37.404	1316	477.94	0.00000003
Tmax Spring	1	21.497	1315	456.44	0.00000354
**Walla Walla**					
NULL			1421	497.29	
Proximity	3	63.887	1418	433.40	<0.00001
Crops Adjacent	5	16.716	1413	416.69	0.0050
Species Diversity	3	6.524	1410	410.16	0.08871
Transport	3	17.834	1407	392.33	0.0004759
Summer Precip	1	6.020	1406	386.31	0.0141442
Tmax Winter	1	25.127	1405	361.18	0.00000053
Tmax Spring	1	14.881	1404	346.30	0.0001145

**Fig 4:**
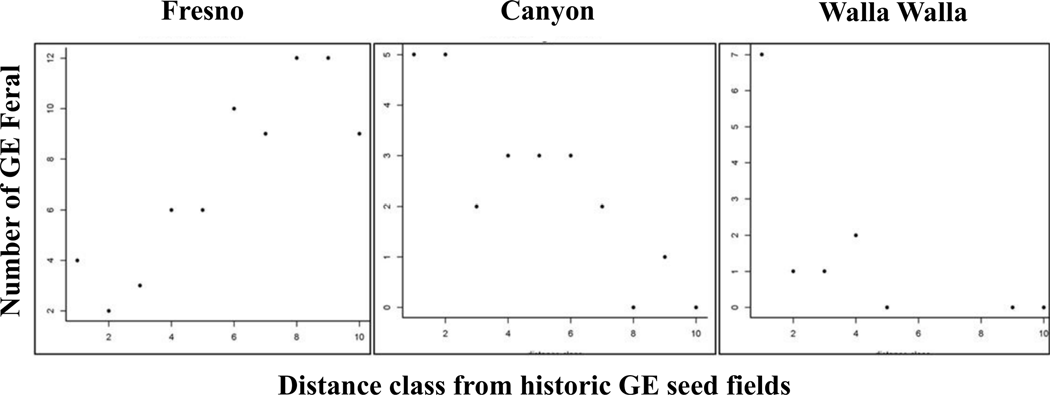
Number of GE feral population occurrences relative to the distance from historic GE seed fields. Relationship was significant but inconsistent across counties. In Fresno County, transgenic feral populations occurred more frequently at further distances from the single historic seed field, while in Canyon and Walla Walla counties, transgenic populations occurred closer to historic GE seed fields.

In all three study areas we found the transgene in feral populations (Figs 1–3). Across study areas, 20.5% of the populations contained the transgene (calculation based on random sites). In ten populations where individual plants were tested, we found three populations that had all negative plants, two populations that were all positive and four populations that contained positive and negative plants, although the populations were heavily biased one way or the other. Incidence of transgene dispersal varied with location, with Fresno County having the highest incidence, and Walla Walla County the lowest (Table 3). The limited numbers of transgenic feral populations precluded a detailed spatial analysis. However, the results of the generalized linear model indicated that the following variables were significant in explaining the occurrence of transgenic feral populations (Table 5). Spillage (during production and transport) was significant in Fresno and Canyon counties. Transgenic feral plants were consistently found at locations where the probability of seed escape was high, such as adjacent to original GE seed fields, or on roads used to transport GE seed to conditioning plants. The total number of feral plants at a site was significant for Fresno and Canyon counties; the transgene was more prevalent in larger populations of feral plants. Distance to historic GE seed fields was also significant but inconsistent across counties (Fig 4). In Fresno County, transgenic feral plants tended to occur more frequently further away from the single historic seed field, while in Canyon and Walla Walla counties, transgenic plants tended to be more frequent, closer to historic GE seed fields. Using available data on historic GE hay-field locations (expressed as four distance classes (see Table 2), we were unable to test for a distance relationship between feral plants and historic GE hay fields since 80% of our feral plant locations fell into the same distance class, (i.e. 1.6 to 8 km from closest historic GE hay field). Looking at the distance classes for transgenic feral locations, 100%, 96%, and 66% of locations were less than 8 km from the closest GE hay field in Fresno, Canyon and Walla Walla counties, respectively. GLM analysis based on known geographic coordinates for three GE hay fields in Fresno County showed no statistically significant relationship with proximity to transgene positive feral locations.

**Table 5:** Logistic regression model for the influence of spillage during production and transport, population size, and proximity to historic GR hay and seed fields on the occurrence of transgenic roadside alfalfa plants.

Parameter	Estimate	Standard Error	z value	Pr(>|z|)
**Fresno**				
Intercept	-2.038	0.79	-2.576	0.00998
Spillage	-0.528	0.2103	-2.628	0.00859
Sqrt(Total Plants)	0.3149	0.1381	2.281	0.02255
RRA Hay Field	0.00000879	0.0000218	0.402	0.68747
RRA Seed Field	0.0000977	0.0000244	4.005	0.000062
**Canyon**				
Intercept	-1.084	0.972	-1.115	0.26779
Spillage	-0.0626	0.255	-2.450	0.01616
Sqrt(Total Plants)	0.1443	0.0715	2.017	0.04659
RRA Hay Field	0.0001	0.0000445	2.263	0.02600
RRA Seed Field	-0.00014	0.0000533	-2.707	0.00808
**Walla Walla**				
Intercept	-0.3367	1.19277	0.282	0.7777
Spillage	-0.1095	0.43911	-0.249	0.80297
Sqrt(Total Plants)	0.10973	0.15568	0.705	0.48090
RRA Seed field	-0.00051	0.000142	-3.618	0.000297

## Discussion

Within our study areas, the overall occurrence of feral plants was relatively rare. Across the study areas, 4.4% of our random sites had feral populations. Bagavathiannan et al. [38] reported a prevalence of roadside plants in Manitoba that was 2–42 times greater than what we observed, depending on location, but noted that alfalfa had been historically planted along roadsides to control erosion. In our study area, only Canyon County used alfalfa in revegetation seed mixes, but only prior to 2000 (Cathy Ford, personal communication, 2014). A common concern supported by modelling, is that transgenic roadside plants may act as conduits to facilitate transgene flow [38, 48]. Supporting evidence would include the spatial distribution of populations within pollinator range, the occurrence of mixed-age populations, evidence that populations may be self-sustaining, and the presence of negative plants with transgenic seed (evidence for current year pollen transfer). In our study, mean distance between nearest neighboring populations in all three study areas (845 m, Fresno County; 1318 m, Canyon County; 812 m, Walla Walla County) fell well within foraging ranges reported for honey bees (i.e. 745–1413 m [49] to 9.5 km [50]), leaf cutter bees (generally < 1600 m [51, 39]), and alkali bees (average 1.6 km, but have been found up to 11 km from their nest sites [52, 53]). The distance where spatial autocorrelation was significant was also well within pollinator foraging range. Almost half of the feral populations included mixed age plants and demographic surveys that tested individual plants within 10 mixed-age populations showed half of the populations were mixtures of positive and negative plants. Although this suggests populations may be self-sustaining, further research is needed to rule out the ingress of seed at more than one time. However, Bagavathiannan et al. [38] reported that roadside feral populations in Manitoba reproduced successfully. We also observed instances where plants tested negative for the transgene, but seed was positive (one plant in Fresno County, nine in Canyon County and two in Walla Walla County). Transgene flow in feral populations has been confirmed in other GE crops such as oilseed rape (*Brassica napus*) [14]. Our results supported evidence that feral transgenic plants could spread transgenes to neighboring feral plants, and potentially to neighboring non-GE fields. Further research is needed to confirm that feral populations are self-sustaining, estimate the frequency of transgene flow and assess the consequences of varying levels of AP in non-GE seed fields resulting from feral transgene movement.

The frequency of sites having transgenic feral plants varied among our study areas. Transgenic plants were found in 32.7%, 21.4% and 8.3% of feral plant sites in Fresno, Canyon and Walla Walla, respectively. Despite having only a single historic GE seed field (64 ha), Fresno County had almost twice as many transgenic feral sites than the next highest area. California state law mandates the use of least toxic herbicides, and the post emergent spray used on county roadsides is glyphosate (Mike Konda, personal communication, 2014). In Canyon County and Walla Walla County roadside sprays contain a mixture of glyphosate and 2,4,D (Jim Martel, Cathy Ford, personal communication, 2014). Of the six feral populations in Fresno County where we tested individual plants, and found both positive and negative plants, four of the populations contained only 1 or 2 negative plants. Further research is needed to confirm if roadside sprays of glyphosate have inadvertently selected for transgenic roadside populations in Fresno, resulting in the relatively high presence of transgenic feral plants, despite the occurrence of only a single historic GE-seed field.

Although climate, road verge habitat, and local cropping pattern were associated with the occurrence of feral plants, they did not have predictive value. There may be several explanations for this. First, as a non-native plant escaping from cultivation, alfalfa's occurrence is likely to be influenced by anthropogenic activities, ecological requirements and interactions with native flora and fauna, making it difficult to identify what specific variables account for occurrence. In explaining the origins of feral oilseed rape populations, Pivard et al. [48] reported similar challenges in separating the influence of human versus environmental explanatory variables that may influence the occurrence of roadside populations. Second, the rarity of feral plants made it difficult to discern the relation between occurrence and local conditions because few “present” data were available compared to the high number of “absent” data. In hindsight, the use of an adaptive sampling strategy that took into account sampling a rare event would have strengthened our analysis. Despite having relatively few sites that contained feral plants, when we examined the relation between transgenic feral plants and our explanatory variables, seed spillage during production and transport did predict the occurrence of transgenic feral plants. Seed spillage occurring during production or transport has been identified as an important factor contributing to the dispersal of transgenes into the environment for GE oilseed rape, another crop with a high level of ferality [12, 10, 54, 55, 56]. Although we were unable to identify specific local conditions that influence the occurrence of feral plants, knowing that feral plants are most likely to occur in alfalfa hay and seed production areas narrows the geographic area where feral plant control should occur.

To our knowledge, our study is the first to confirm that alfalfa has joined oilseed rape as a genetically engineered crop that has dispersed beyond cultivated fields. Assessing the risk that transgenic feral plants contribute to AP in conventional fields requires an evaluation of exposure (i.e., how frequent is the occurrence of transgenic feral plants?) and consequence (i.e., are AP levels in conventional seed fields negatively impacted by feral transgene flow?), and our study has provided empirical evidence on the relative frequency of transgenic feral plants in three important alfalfa production areas. Our data suggest that these populations are self-sustaining and that gene flow is likely. Still to be determined is the ecological and economic consequences of transgenic feral alfalfa plants. It seems unlikely that the low levels of transgenic populations we observed in 2011 and 2012 could cause measurable levels of adventitious presence (i.e. ≥ 0.1% AP) in conventional seed lots. However, if grower adoption rates match those of GE cotton, soybean, corn and oilseed rape, the occurrence of transgenic feral populations will increase, and negative consequences may become evident at some point. To ensure the coexistence of alfalfa producers targeting GE, non -GE and GE-sensitive markets, best management practices that limit seed spillage and control feral plants along public roadways in alfalfa hay and seed production areas should be supported

## Supporting Information

S1 File Data set used for analysis. (XLSX)
